# A greater involvement of posterior brain areas in interhemispheric transfer in autism: fMRI, DWI and behavioral evidences

**DOI:** 10.1016/j.nicl.2015.04.019

**Published:** 2015-04-30

**Authors:** Elise B. Barbeau, John D. Lewis, Julien Doyon, Habib Benali, Thomas A. Zeffiro, Laurent Mottron

**Affiliations:** a Centre d'Excellence en Troubles Envahissants du Développement de l'Université de Montréal (CETEDUM), Montréal, QC, Canada; b Centre de recherche de l'Institut universitaire de santé mentale de Montréal, Montréal, QC, Canada; c Département de Psychiatrie, Université de Montréal, Montréal, QC, Canada; d Montreal Neurological Institute, McGill University, Montréal, QC, Canada; e Département de Psychologie de l'Université de Montréal & Unité de Neuroimagerie Fonctionnelle (UNF), Montréal, QC, Canada; f Laboratoire d'Imagerie Fonctionnelle — U678, Faculté de Médecine Pierre et Marie Curie — Pitié Salpétrière, Paris, France; g Neural Systems Group, Massachusetts General Hospital, Psychiatry, Charlestown, MA, USA

**Keywords:** Corpus callosum, Poffenberger, Purdue, Visuo-motor integration, Autism, Cortical reorganization

## Abstract

A small corpus callosum (CC) is one of the most replicated neurobiological findings in autism spectrum (AS). However, its effect on interhemispheric (IH) communication is unknown. We combined structural (CC area and DWI), functional (task-related fMRI activation and connectivity analyses) as well as behavioral (Poffenberger and Purdue tasks) measures to investigate IH integration in adult AS individuals of typical intelligence. Despite similar behavioral IH transfer time and performances in bimanual tasks, the CC sub-regions connecting frontal and parietal cortical areas were smaller in AS than in non-AS individuals, while those connecting visual regions were similar. The activation of visual areas was lower in AS than in non-AS individuals during the presentation of visual stimuli. Behavioral IH performances were related to the properties of CC subregions connecting motor areas in non-AS individuals, but to the properties of posterior CC regions in AS individuals. Furthermore, there was greater functional connectivity between visual areas in the AS than in the non-AS group. Levels of connectivity were also stronger in visual than in motor regions in the autistic subjects, while the opposite was true for the non-autistic group. Thus, visual IH transfer plays an important role in visuo-motor tasks in AS individuals. These findings extend the well established enhanced role of perception in autistic cognition to visuo-motor IH information transfer.

## Highlights

• The size of the corpus callosum connecting the motor region is reduced in autism.
• The interhemispheric transfer of visuo-motor information is not impaired in autism.
• In autism, the posterior corpus callosum is more involved than the motor sections.
• Plastic reorganization in autism leads to atypical structure–function relationship.
• The results agree with a greater involvement of perceptual brain areas in autism.

## 1 Introduction

The observation that the corpus callosum (CC) is smaller in autism spectrum (AS) individuals than in non-AS individuals is among the most replicated neurobiological findings in AS. A meta-analysis of structural studies (Frazier and Hardan, 2009), two reviews of diffusion tensor imaging (DTI) studies (Travers et al., 2012; Vissers et al., 2012) and one meta-analysis of diffusion tractography studies (Aoki et al., 2013) support the conclusion that structural connectivity is altered in the CC of AS individuals. Early investigations examining alterations of functional connectivity in AS individuals found evidence of low intrahemispheric, fronto-posterior long-distance connectivity (Schipul et al., 2011; Uddin et al., 2013; Vissers et al., 2012), associated with short distance (or local) over-connectivity (Just et al., 2004; Just et al., 2007). Recent reconsideration of these findings emphasizes the dependence on the methodology used of conclusions drawn from functional connectivity (Muller et al., 2011) and anatomical CC (Lefebvre et al., 2014) investigations in AS populations.

Several other studies have observed higher connectivity in AS involving perceptual areas, regardless of the distance (Di Martino et al., 2011; Dominguez et al., 2013; Keown et al., 2013; Léveillé et al., 2010; Shen et al., 2012; Supekar et al., 2013). Besides alterations of CC volume, alterations of intrahemispheric frontal, temporal and parietal white matter volume (Amaral et al., 2008; Anagnostou and Taylor, 2011; Just et al., 2012) and diffusion properties (Aoki et al., 2013; Travers et al., 2012; Vissers et al., 2012) also suggest that widespread alterations of connectivity occur in AS.

The CC is the main white matter bundle connecting the two brain hemispheres; therefore, morphological and microstructural alterations of this brain region should negatively influence interhemispheric (IH) connectivity. EEG signal coherence between bilateral frontal and temporoparietal regions (Carson et al., 2014; Catarino et al., 2013; Coben et al., 2008; Lazarev et al., 2015) and between bilateral visual regions (Clawson et al., 2015; Isler et al., 2010; Lazarev et al., 2015) is lower in AS than in non-AS individuals. In addition, MRI functional connectivity studies also report that IH connectivity is impaired between several bilateral frontal regions (Verly et al., 2014) and between sensorimotor frontal areas, frontal and parietal superior insula, and temporal and inferior premotor areas in AS individuals (Anderson et al., 2011). Alterations of functional connectivity are related to anomalies in the microstructural properties (McGrath et al., 2013) and reduction in size (Cherkassky et al., 2006; Damarla et al., 2010; Just et al., 2007; Kana et al., 2006; Mason et al., 2008) of the CC. The size of the CC in AS individuals is also correlated with their neurocognitive performance (Alexander et al., 2007; Keary et al., 2009) as well as with the number and magnitude of autistic signs (Billeci et al., 2012; Cheung et al., 2009; Hanaie et al., 2014).

Direct evidence supporting a link between atypical structure, IH information transfer and behavioral performance is nonetheless lacking and the effect of alterations of CC size on the speed and efficiency of IH communication remains to be investigated. Information transfer between brain hemispheres can be investigated by a simple reaction time paradigm in response to lateralized visual stimuli: the Poffenberger paradigm (Poffenberger, 1912), which gives a measure of IH transfer time. The relevant variable for this measure is the Crossed/Uncrossed Difference (CUD), which is obtained by subtracting the manual response time (button press) to a visual stimulus presented in the uncrossed circuit (i.e. the response of the hand ipsilateral to the stimulated visual hemifield) from that of the crossed circuit (i.e. the response of the hand contralateral to the visual hemifield). Uncrossed trials can be processed by visual and motor areas of the same hemisphere, whereas crossed trials necessitate a transfer of information from one hemisphere to the other. The CUD therefore reflects the time needed for the visuo-motor information to cross from one hemisphere to the other via the CC. The Poffenberger paradigm has been used in populations of non-AS individuals, in conjunction with fMRI, to identify the motor and visual cortical areas involved in information transit through the CC. These studies have revealed that the CUD is correlated with the signal intensity difference between the crossed and the uncrossed circuits (Iacoboni and Zaidel, 2004; Omura et al., 2004). The Poffenberger paradigm has never been applied to AS populations, either in behavior tests or in neuroimaging studies. Behavioral performance under the dependence of IH communication can also be assessed by bimanual coordination tasks. The Purdue pegboard is a test that examines gross and fine motor skills. It comprises two bimanual conditions requiring the coordinated movement of both hands to rapidly place little pieces into the pegboard in a simultaneous or sequential manner. This task also requires hand–eye coordination. Bimanual motor skills are dependent on the integrity of the CC (Johansen-Berg et al., 2007) and are affected in people with CC agenesis (Badaruddin et al., 2007).

The aim of this study was to establish whether the alterations of the size of the CC observed in AS affect IH transfer, and whether the cortical areas involved in IH information transfer differ in AS and non-AS individuals. We used anatomical, functional connectivity indices, and diffusion weighted MRI (DWI) of the CC, coupled with two measures of IH transfer, the Poffenberger paradigm and the Purdue pegboard task, to investigate IH integration. IH transfer was expected to be slower in AS than in non-AS individuals, because CC size is thought to affect the speed of IH transfer. However, variability in the allocation of cortical regions involved in visuo-motor tasks in AS individuals (e.g. Pierce et al., 2001; Poulin-Lord et al., 2014) as well as the use of plastic cortical rededication (see Mottron et al., 2014 for review) may result in anatomical brain differences without deleterious consequence on IH transfer.

## 2 Materials and methods

### 2.1 Participants

Thirty-four AS and 33 non-AS individuals aged between 14 and 37 years old participated in this study. Participants were randomly recruited from the research database of the Specialized Autism Clinic at the Rivière-des-Prairies Hospital (Montreal, Canada). Exclusion criteria for all participants were uncorrected visual impairment, the use of drugs or alcohol exceeding two drinks per day, and a Full Scale Intellectual Quotient (Wechsler FSIQ) score inferior to 75. Two AS participants took medication (one quetiapine, one methylphenidate). Twenty-seven out of 34 AS participants were diagnosed by the Autism Diagnostic Interview-Revised (ADI-R; Lord et al., 1994) and the Autism Diagnosis Observation Schedule module 3 or 4 (ADOS-G; Lord et al., 2000), combined with an expert interdisciplinary clinical assessment. Seven participants were characterized according to expert interdisciplinary judgment alone (one participant) or in combination with either the ADOS-G (two participants) or the ADI-R (four participants). None of the AS participants had comorbid genetic, neurological, or DSM-IV Axis 1 psychiatric conditions, except for hyperactivity and language disorders, which are present in a large proportion of AS individuals at some point during their development. All AS participants received a DSM-IV diagnosis of autism and presented speech onset delays and/or atypicalities. The terms autism or autistic will therefore be used to refer more specifically to the AS population under study. Non-AS participants were screened through a questionnaire to exclude individual or familial neurological, psychiatric, or medical conditions known to affect brain function.

Handedness was measured with the Edinburgh inventory (Oldfield, 1971) and the Hand Preference Demonstration Test (Soper et al., 1986). The scores of these two measures were consistent for all participants. Handedness affects CC size and function in typically developing populations (Witelson, 1985, 1989), moreover, left and right-handed people display different patterns of IH transfer time with the left and the right hand (Marzi et al., 1991); therefore, only right-handed people were included in this study.

Written informed consent was obtained from all participants in accordance with the Regroupement Neuroimagerie/Québec IRB approved protocol 08-09-003 and the research ethics committee of the Rivière-des-Prairies Hospital, Montréal, Canada. All participants received compensation for their participation. Autistic and non-AS groups were comparable in terms of sex, age (14–37 years old) and handedness (Edinburgh: 17–100). Because the documented relationship between CC size and IQ is mostly driven by PIQ (Ganjavi et al., 2011), intelligence matching was achieved using non-verbal IQ (PIQ: 77–127, RPM percentile: 10–100). Raven IQ is more representative of autistic intelligence (Barbeau et al., 2013), the later being underestimated by FSIQ measures (Dawson et al., 2007; Nader et al., 2014). Matching on FSIQ could also bias AS samples towards larger brain volumes, which is thought to affect the group differences in CC size (Lefebvre et al., 2014). A subsample of 22 AS and 24 non-AS MRI-compatible individuals, matched according to the same variables as the overall group, was included in the MRI part of the experiment. Table 1 shows the socio-demographic characteristics of the participants. One AS and two non-AS participants were excluded because of artifacts in the data associated with a large amount of head motion. One more participant in each group was removed because of their performance in the tasks (see Data analysis section). The final group included in the fMRI second level analysis comprised 20 AS and 21 non-AS individuals matched for PIQ, Raven percentile and age.

### 2.2 Stimuli, apparatus and procedure

#### 2.2.1 The Poffenberger task

##### 2.2.1.1 Behavioral task outside the scanner

**Fig. 1:**
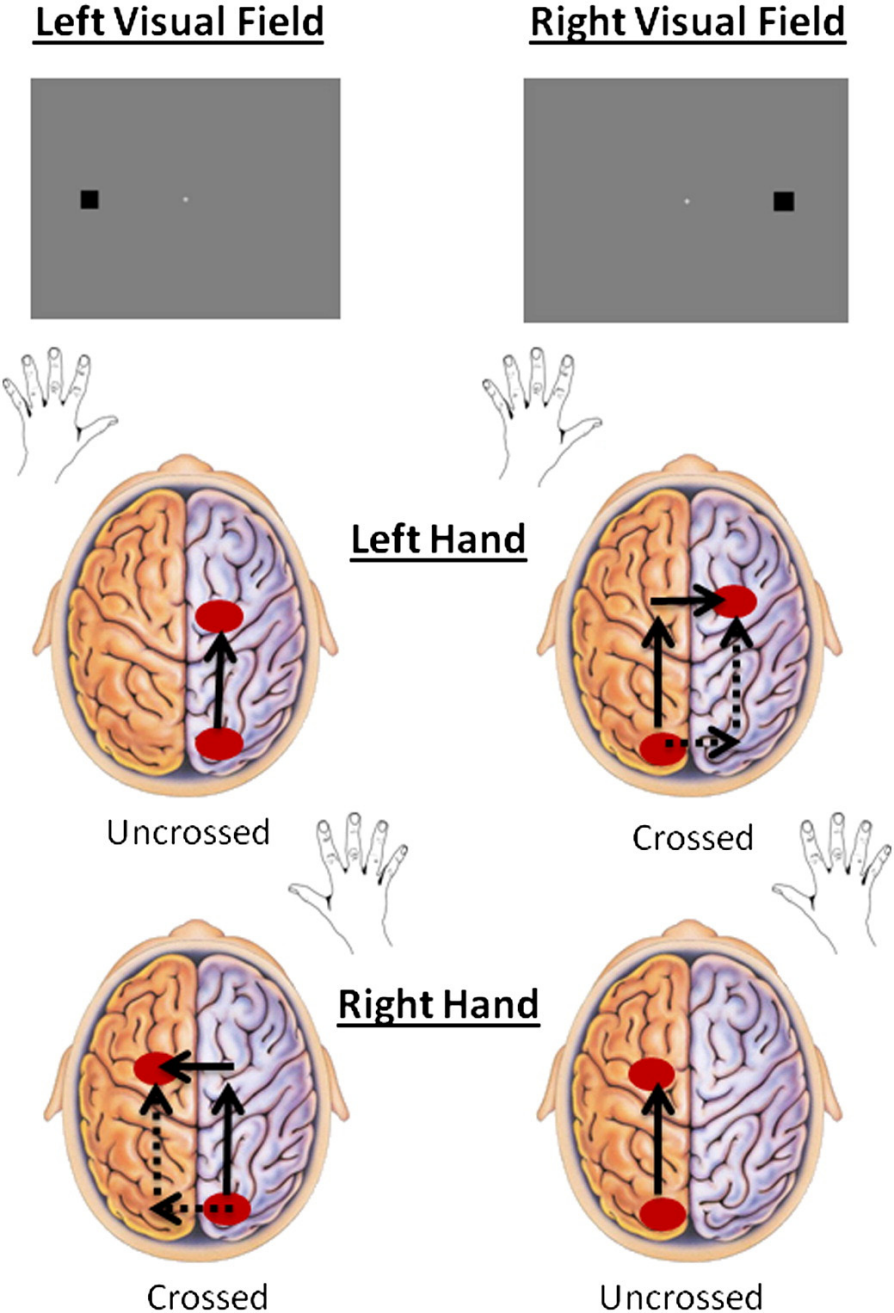
Stimulus presentation in the left visual field (LVF) and in the right visual field (RVF).

The task consisted of detecting a black square that randomly appeared on the gray background either to the right or to the left of a central fixation cross (Fig. 1). The black stimulus had an eccentricity of 8°. In each experimental block, subjects were presented with 50 stimuli to the right and 50 to the left of the central fixation cross. Each stimulus lasted 50 ms, which is shorter than a visual saccade (~200 ms: Rayner, 1998). This ensured that each stimulus was presented to only one cerebral hemisphere. The interstimulus interval varied randomly from 1000 to 3500 ms to avoid anticipation. The testing session consisted of three right and three left hand blocks (each lasting about 4 min) of 100 trials each, for a total of 600 trials. The order of blocks varied for each participant and was balanced across groups. There were 150 trials in each of the four conditions (Left hand–Left visual field: LH–LVF, Left hand–Right visual field: LH–RVF, Right hand–left visual field: RH–LVF, Right hand–Right visual field: RH−RVF). A practice block of 16 trials (eight left and eight right stimuli) for each hand was administered before the task.

The participants were seated in a quiet and dimly lit room with black wooden panels on each side and one in front with an opening for the computer screen, to minimize visual distraction. A chin rest minimized head movement and maintained the viewing distance at 73 cm from the screen. The response box was placed either to the right or left of the participants to ensure a 90° angle of the responding arm. Participants were instructed to keep fixating their eyes on the cross throughout the task, and to press the button as quickly as possible with one of their index fingers every time they saw a black square, regardless of the side on which it appeared.

The experiment was carried out with *E-prime* software Version 1.2 (Psychology Software Tools Inc.) on a 19 in. CRT monitor, with a 120 Hz refresh rate. Monitor luminance was checked with a photometer before each session. The manual response was recorded with the PST Serial Response box that has a 0 ms debounce period.

#### 2.2.2 Behavioral task inside the scanner

The Poffenberger paradigm was adapted to suit to a single-event paradigm and to favor high levels of blood oxygen level dependent (BOLD) activation in the visual cortex. Easily discernible, black and white checkerboard stimuli were presented on a gray background, and presentation time was increased to 100 ms, which is still shorter than a visual saccade. The interstimulus intervals (ISIs) were longer than in the task outside the scanner, which allowed the hemodynamic response to return to baseline between trials. The ISI was varied pseudo-randomly between 5000 and 12,000 ms as follows: 6 × 5000 ms, 5 × 6000 ms, 4 × 7000 ms, 3 × 8000 ms, 1 × 10,000 ms, 1 × 11,000 ms, and 1 × 12,000 ms, with an average of about 7000 ms. In motor related areas, fMRI activation decays linearly over time during the repeated execution of motor response paradigms (Mancini et al., 2009); therefore, an event related design was chosen to optimize the detection sensitivity. Long ISIs improve the sensitivity of the signal (Price et al., 2006) and reduce predictability. There were 84 trials per block and four blocks (two with each hand) lasting about 9 min each. Before each block, participants were told which hand they should use throughout the block, and were instructed to press the button as fast as possible with the index finger as soon as they saw the stimulus. The participants were lying in a supine position in the scanner, and held an MRI-compatible Fiber Optic Response Pad (Current design INC.) in each hand. The response box was connected to a computer equipped with a parallel port to improve the accuracy of timing. Visual stimuli were presented with *E-prime*, Version 2.0, and were presented to participants though a mirror installed on the head coil, which allowed them to see the stimuli on a screen installed at the back of the scanner.

Measurements of the Poffenberger effect in the scanner are less accurate than those outside the scanner due to the resolution of the MRI-compatible response box and screen, the suboptimal (less ergonomic) position of the participants arms which may have affected the participant's response times, and the presence of distractors (e.g. noise, immobility). Moreover, it has been suggested that brain processes are slower under the influence of the magnetic field (Foucher et al., 2008). Although both tasks yielded similar results, only the task outside the scanner was considered for behavioral analysis.

#### 2.2.3 The Purdue pegboard test

The Purdue pegboard test (Model 32,020, Lafayette Instrument Co., IL, USA) assesses fine and gross motor skills, dexterity and coordination and was performed outside the scanner. It has two bimanual conditions in which participants have to 1) move and place in small holes as many small pegs as possible within 30 s with both hands (BH condition) working simultaneously, and 2) assemble washers, collars, and pegs on the pegboard in a specific sequence using both hands in a coordinated and sequential fashion within 60 s (Assembly condition). Each condition is completed three times and averaged. Absolute performance in the bimanual conditions of this task (Purdue BH and Assembly measures) as well as the relationship between these variables (number of pegs placed in 30 s) and the properties of the CC were relevant measures for this study.

#### 2.2.4 MRI image acquisition

Images were acquired on a 3 T Siemens Tim Trio scanner with a 32 channel phased-array head coil at the “Unité de Neuroimagerie Fonctionnelle” (University of Montreal). The scanning session included an anatomical T1-weighted structural brain image obtained with an ME-MPRAGE 4-Echo sequence (176 slices, 1 mm^3^ voxels, TR = 2530 ms, TE = 1.64/3.5/5.36/7.22 ms, flip angle = 7°), which has a low distortion and high signal-to-noise ratio (van der Kouwe et al., 2008). Functional data were acquired with an echo planar imaging (EPI) pulse sequence (150 acquisitions, TR = 3330 ms, 60 slices, matrix size 80 × 80 voxel size 2.5 × 2.5 × 2 mm^3^, slice thickness: 2 mm with a 0.5 mm gap, TE = 30 ms, flip angle = 90°). Gradient echo phase and magnitude field maps were then acquired (60 slices, matrix size = 80 × 80, voxel size 2.5 × 2.5 × 2.0 mm^3^, slice thickness = 2 mm with a 0.5 mm gap, TR = 488 ms, TE short = 4.92 ms, TE long = 7.38 ms, flip angle = 60°) for the correction of image distortions and the improvement of co-registration accuracy with the field map toolbox in SPM. Diffusion weighted images (DWI) were acquired with an echo-planar sequence (TR = 8740 ms; TE = 83 ms; 70 axial slices; FOV 256 mm; matrix = 128 × 128; 2 mm interleaved slices; 128 directions; b values = 0 and 700 s/mm^2^). Field maps matched to the diffusion-weighted images were also acquired.

### 2.3 Data analysis

#### 2.3.1 Behavioral task

Trials with a response time (RT) under 150 ms or above 500 ms (outside the scanner) or 800 ms (inside the scanner), for a total of 2.3% of trials, were considered as commission and omission errors, respectively and were removed. Median RT was computed for each of the four conditions (LH–LVF, LH–RVF, RH–LVF, RH–RVF) and for each participant. A repeated measure ANOVA was conducted with Hand (left and right) and Visual field (left and right) as within factors and Group as a between factor. The Crossed–Uncrossed Difference (CUD) was individually computed by subtracting the RT of the two uncrossed conditions from the RT of the crossed conditions of both hands. Participants within each group with CUD values >2 SD from the group average were considered as outliers and removed from the analysis. For trials outside the scanner, two participants in each group were excluded to give a final total of 32 AS and 32 non-AS individuals. For trials inside the scanner, one participant in each group was excluded because they missed more than 20% of the trials and another participant in each group was excluded because they had an outlier CUD value.

#### 2.3.2 T1 structural and DWI image analysis

The T1-volumes were processed with CIVET, a fully automated structural image analysis pipeline developed at the Montreal Neurological Institute. CIVET corrects intensity non-uniformities by non-parametric non-uniform intensity normalization (N3: Sled et al., 1998); aligns the input volumes to the Talairach-like ICBM-152-nl template, with an affine transformation followed by a non-linear transformation (Collins et al., 1994); classifies the input volumes into white matter, gray matter, cerebrospinal fluid, and background (Zijdenbos et al., 2002); and extracts the white-matter and pial surfaces (Kim et al., 2005). The CIVET non-linear transformation was then refined with the minctracc program, and the result was used to warp a parcellated (25 subregions) template of the CC, defined on the ICBM-152-nl template, to overlay each subject's T1-volume. This procedure ensured that subregions of the CC were comparable among participants. The size of each CC subregion was then measured and this value was divided by the surface area of the portions of cortex connected via each CC subregion to provide a measure of the size of each CC region relative to the amount of gray-matter it connects (RelCC). The surface atlas was produced from an independent sample in which probabilistic tractography was used to map surface vertices to CC subregions (the procedure is illustrated in Supplementary Fig. 1 and described in detail in Lewis et al., 2013). This surface atlas was registered to each participant to provide the measures of cortical surface area connected by each subregion of the CC. The 25 CC subregions, numbered from anterior to posterior, were then divided into five different groups to investigate the properties of the CC connecting particular functionally related cortical areas: CCs 1–3 (prefrontal cortical areas), CCs 4–9 (frontal areas), CCs 10–16 (para-central areas), CCs 17–21 (parietal areas), and CCs 22–25 (occipital areas).

**Fig. 2:**
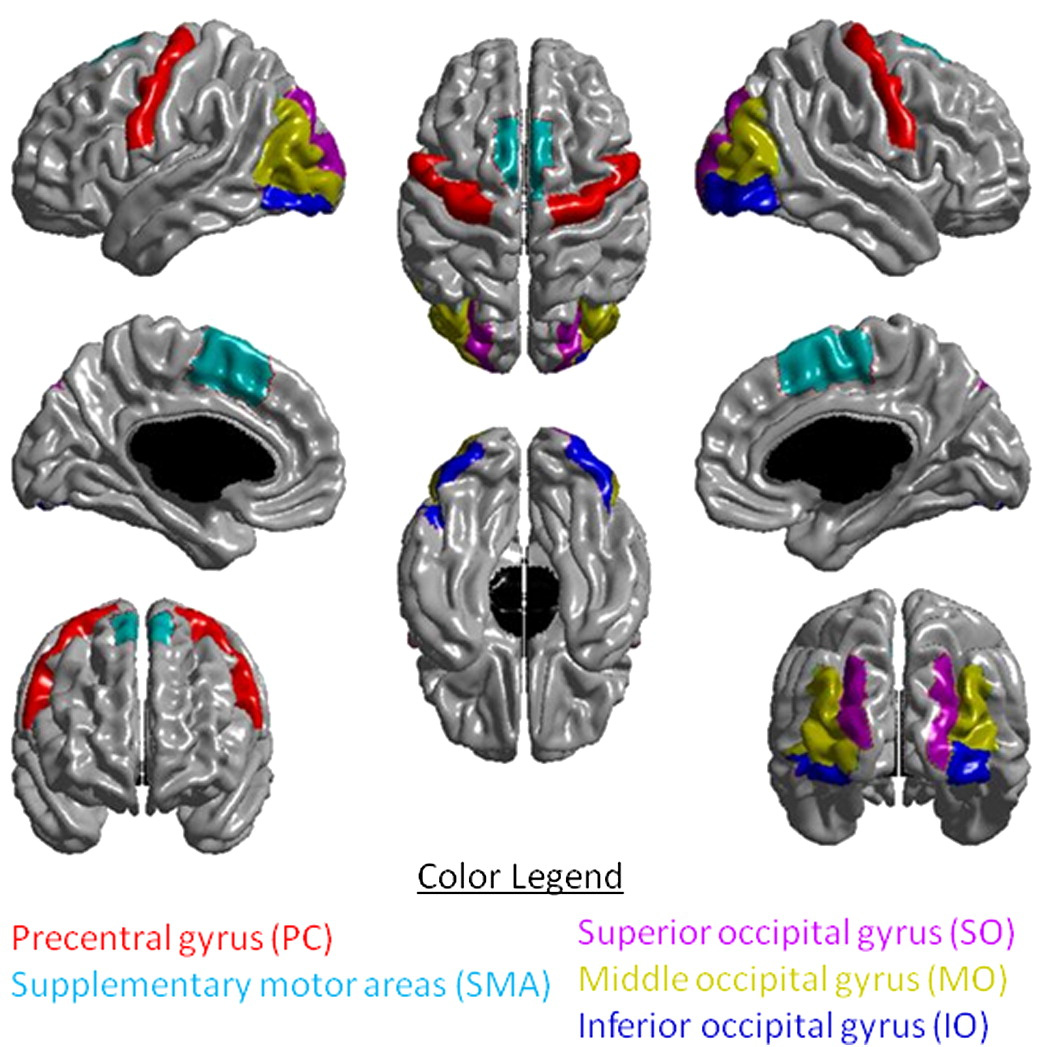
The five bilateral AAL regions of interest used as target masks for probabilistic tractography.

Field maps were used to correct the diffusion-weighted images for distortions caused by inhomogeneities in the magnetic field and the images were converted to 4D volumes. The diffusion volumes were motion corrected, cleaned of artifacts, scaled to stereotaxic spcae, and unwarped to structural volume. The resulting volumes were processed with FSL's *dtifit* to calculate fractional anisotropy (FA), axial diffusivity (AD), radial diffusivity (RD) and mean diffusivity (MD), and with *bedpostx* to calculate the orientation distribution function at each voxel. The T1 was registered with the b0 diffusion volume, which allowed diffusion-based measures to be calculated for each CC subregion, and provided the transformation necessary to carry out probabilistic tractography with masks derived from the T1 volume. The CIVET results were used to construct the seed, stop, and target masks for use with FSL's *probtrackx* (Behrens et al., 2007). Tractography is seeded 10000 times in each white-matter voxel. Stop masks determine where tract propagation is halted; stop masks were voxels on the boundary of white-matter, including the ventricles and subcortical gray matter. Target masks determine the mapping from voxels of the stop masks to brain regions; target masks were the voxels at the boundary of white matter and the cortex in the following five bilateral regions of interest of the Automatic Anatomical Labeling (AAL) atlas (Tzourio-Mazoyer et al., 2002): the precentral (PC) gyrus, the supplementary motor area (SMA), the superior occipital (SO) gyrus, the middle occipital (MO) gyrus, and the inferior occipital (IO) gyrus (Fig. 2). FSL's *dtifit* was used to produce FA, AD, RD and MD volumes. These were overlayed on probabilistic connectivity maps for the IH connections for each set of homeotopic pairs (see Supplementary Fig. 2), and for each one, the weighted mean was computed for each of the diffusion masures.

#### 2.3.3 Statistical analysis

The values of relative corpus callosum (RelCC) areas and the DWI measures superior or inferior to 2 SD from the group mean were removed (an average of 4.8% of data were removed in the AS group and 3.6% in the non-AS group). Multivariate analyses of variance (MANOVA) were conducted to investigate whether the CC measures differed between groups. There was no significant effect of age or intelligence measures when entered as covariates, and thus they were not included in the final model.

#### 2.3.4 fMRI image analysis

##### 2.3.4.1 Preprocessing

SPM8 was used for preprocessing and statistical modeling. During preprocessing, images were realigned and unwarped, corrected for slice timing, coregistered to anatomical scans, segmented into gray matter, white matter and CSF, and then spatially normalized into the ICBM152 MNI space. Normalized images were finally smoothed with a 3-D Gaussian filtering kernel of 8 mm FWHM.

##### 2.3.4.2 Statistical modeling

First-level analyses for each subject were conducted with a design matrix for each of the four blocks including the two visual field conditions (left/right) as conditions of interest. Missed trials were entered as a condition of non-interest to exclude any effects related to them. A high-pass temporal filter with a cutoff of 128 s was used to remove low-frequency noise. The hemodynamic response was modeled with the canonical hemodynamic function implemented as boxcar basis functions in SPM8.

In the first-level analysis, contrasts were computed for the four conditions: Left hand–left visual field (LH–LVF), Left hand–right visual field (LH–RVF), Right hand–left visual field (RH–LVF) and Right hand–right visual field (RH–RVF) vs. the fixation cross baseline. Second-level analyses were then performed to allow inferences about the population by entering the first-level contrasts for each condition in a flexible factorial model with Subject (41 levels), Group (two levels, unequal variance) and Condition (four levels, equal variance) as factors.

Contrasts were computed to isolate the activity specific to each responding hand (LH minus RH: LH–LVF and LH–RVF vs. RH–LVF and RH–RVF, RH minus LH: RH–LVF and RH–RVF vs. LH–LVF and LH–RVF) and each stimulated visual field (LVF minus RVF: LH–LVF and RH–LVF vs. LH–RVF and RH–RVF, RVF minus LVF: LH–RVF and RH–RVF vs. LH–LVF and RH–LVF) to examine within-group voxel-wise estimates of task-related activity. A critical threshold of *p* < .05, FWE-corrected and extent threshold of 50 voxels were used. Cortical activity peaks were located with SPM8 Anatomy toolbox.

##### 2.3.4.3 Laterality index

The LI Toolbox (Wilke and Lidzba, 2007) was used to compute laterality indexes (LIs) with measures of voxel count and voxel value in each hemisphere to investigate whether the groups differed in terms of the magnitude of lateralization of motor and visual brain activity. LIs were computed for two motor (BA4 and BA6) and two visual (BA17 and BA18-19) masks defined anatomically with the WFU PickAtlas (Maldjian et al., 2003) using contrasts (hand or visual field conditions) and subject-specific adaptive threshold. The outcome values range from −1 (right lateralization) to +1 (left lateralization). The LI measures were transformed to absolute values to measure the magnitude of the lateralization (and not its direction), with a score of 0 corresponding to no lateralization (equal in both hemispheres), and a score of 1 corresponding to complete lateralization.

#### 2.3.5 Functional connectivity analyses

In order to estimate the levels of functional connectivity between bilaterally activated motor and visual areas, we extracted regions of interest (ROIs) from bilateral group (conjunction) activation contrasts. Hand-specific left and right primary motor cortex ROIs were obtained by contrasting response laterality (MNI coordinates of each cluster's maxima: motor left [−38, −28, 64], motor right [38, −24, 66]). Two visual cluster per hemisphere were also used in this analysis based upon left/right visual field contrast (MNI coordinates: visual 1 left [−42, −74, 10], visual 2 left [−36, −72, −14], visual 1 right [48, −68, 10], visual 2 right [22, −70, −12]). Spherical 5 mm radius ROIs were defined from these 6 coordinates. For each participant, averaged ROI voxel time courses were extracted to compute ROI pairwise Pearson correlation coefficients. The correlation coefficients for the two visual ROIs were averaged and entered in a repeated measure ANOVA with Modality as a within-subject factor (2 levels: motor, visual) and Group as a between-subjects factor (2 levels). One extreme data-point in the AS group was excluded for the visual modality as it was more than 3.5 SD below the group average.

### 2.4 Structure–performance relationship

Exploratory regression analysis was conducted to investigate the existence of a relationship between behavioral IH transfer time and Purdue measures and the properties of the CC and to determine whether this relationship differed between groups. A complete model was used to test the relationship between “behavioral measure”, GROUP and their interactions for the various dependent variables (CC measures). Residual normality was tested with the Shapiro–Wilk test and for each model, assumptions (normality, linearity, homoscedasticity) were checked by residual analysis. Assumptions of normality, linearity and homoscedasticity were met.

## 3 Results

### 3.1 Behavioral tasks

#### 3.1.1 Poffenberger

The Poffenberger paradigm was used to investigate the speed of IH transfer of information through the CC. Repeated measures ANOVA revealed a Hand × Field interaction (*F*(1,61) = 19.17, *p* < .001), in which response times (RTs) in the uncrossed trials were faster than in the crossed trials. An independent sample *t*-test showed that the AS and non-AS groups did not differ in terms of IH transfer time (IHTT) measured by the Crossed–Uncrossed Difference (CUD) (AS: *M* = 2.4 ms, *SD* = 5.2, non-AS: *M* = 2.9 ms, *SD* = 4.2, CUD group difference: *t*(61) = −.380, *p* = .705). These measures are in the normal range (1–10 ms; Marzi et al., 1991). The median RT was also similar in each group (AS: *M* = 276.8 ms, *SD* = 47.1, non-AS: *M* = 268.2 ms, *SD* = 27.9, group difference: *t*(61) = .877, *p* = .381). When FSIQ was included in the model as a covariate, it had a significant effect on the CUD measure. However, there were still no significant differences between groups following correction for FSIQ. The exclusion of participants with an IQ <85 (three AS individuals) to match FSIQ between the groups did not affect the results.

**Fig. 3:**
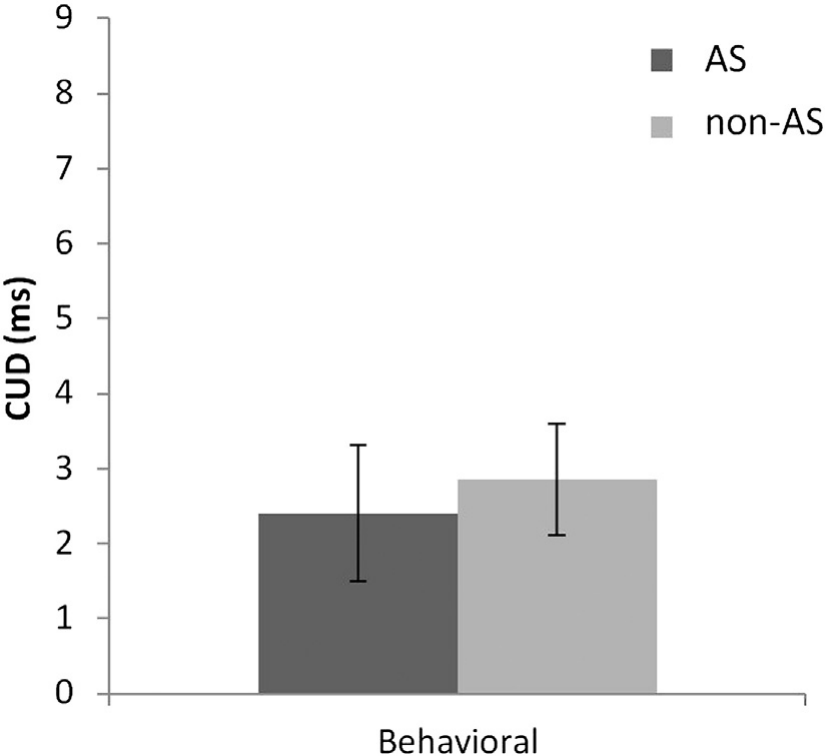
Results of the crossed–uncrossed difference (CUD) in milliseconds (ms) for the autism spectrum (AS) and non-AS groups measured with the Poffenberger task outside (behavioral) the scanner.

The same Hand × Field interaction was obtained in the scanner: RTs in crossed trials were slower than in the uncrossed trials (*F*(1,37) = 20.13, *p* < .001). There was no Group effect but AS individuals tended to have slower CUDs than non-AS individuals (AS: *M* = 6.38 ms, *SD* = 7.77, TYP: *M* = 2.73 ms, *SD* = 4.58, *t*-test: *t*(37) = 1.802, *p* = .080). The median RT was comparable in the AS and non-AS groups (AS: *M* = 408.9 ms, *SD* = 95.4, non-AS: *M* = 421.85 ms, *SD* = 73.3, *t*-test: *t*(37) = −.478, *p* = .635). Results of the Poffenberger task performed outside the scanner are presented in Fig. 3 and those performed in the scanner are shown in Supplementary material (Supplementary Fig. 3).

#### 3.1.2 Purdue

**Fig. 4:**
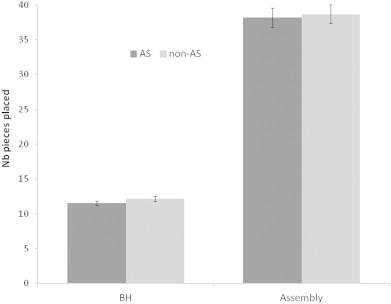
Results of the Purdue pegboard “both hands” (BH) and “assembly” conditions for autism spectrum (AS) and non-autism spectrum (non-AS) participants. The number of pieces placed in 30 (BH) and 60 (assembly) seconds is displayed. Error bars represent standard errors.

For the both hands and assembly conditions, AS participants did not significantly differ from non-AS participants (Fig. 4, and see Barbeau et al., 2015, for detailed statistical analyses and results).

### 3.2 Structural

**Fig. 5:**
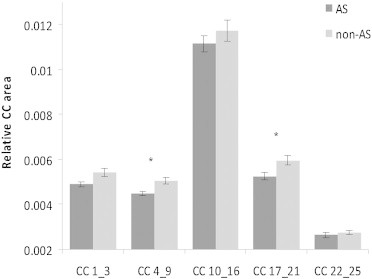
Relative corpus callosum area measures for the subregions 1–3, 4–9, 10–16, 17–21 and 22–25 displayed for the autism spectrum (AS) and non-AS groups. *Group difference *p* < .05.

Total brain volume did not differ between groups (*p* = .455). CC size differed significantly between groups (*F*(1,33) = 3.33, *p* = .015). The relative CC areas (RelCC) connecting frontal (CC4-9; *F*(1,37) = 13.11, *p* = .001) and parietal (CC17-21; *F*(1,37) = 4.71, *p* = .036) cortical regions were significantly smaller in the AS group than in the non-AS group. Structural results are presented in Fig. 5.

We also performed the analysis on the RelCC measures before removal of outliers to account for the large variability in CC size in the population. The results remained unchanged (*F*(1,40) = 2.80, *p* = .029), and the CC in RelCC 4_9 (*p* = .002) and RelCC 17_21 (*p* = .026) regions was still larger in non-AS than in AS individuals.

### 3.3 DWI

No group differences were observed for any of the diffusion metrics (FA, MD, RD, AD) (see Supplementary Fig. 4).

### 3.4 fMRI

#### 3.4.1 Main effects of hand and visual field

**Fig. 6:**
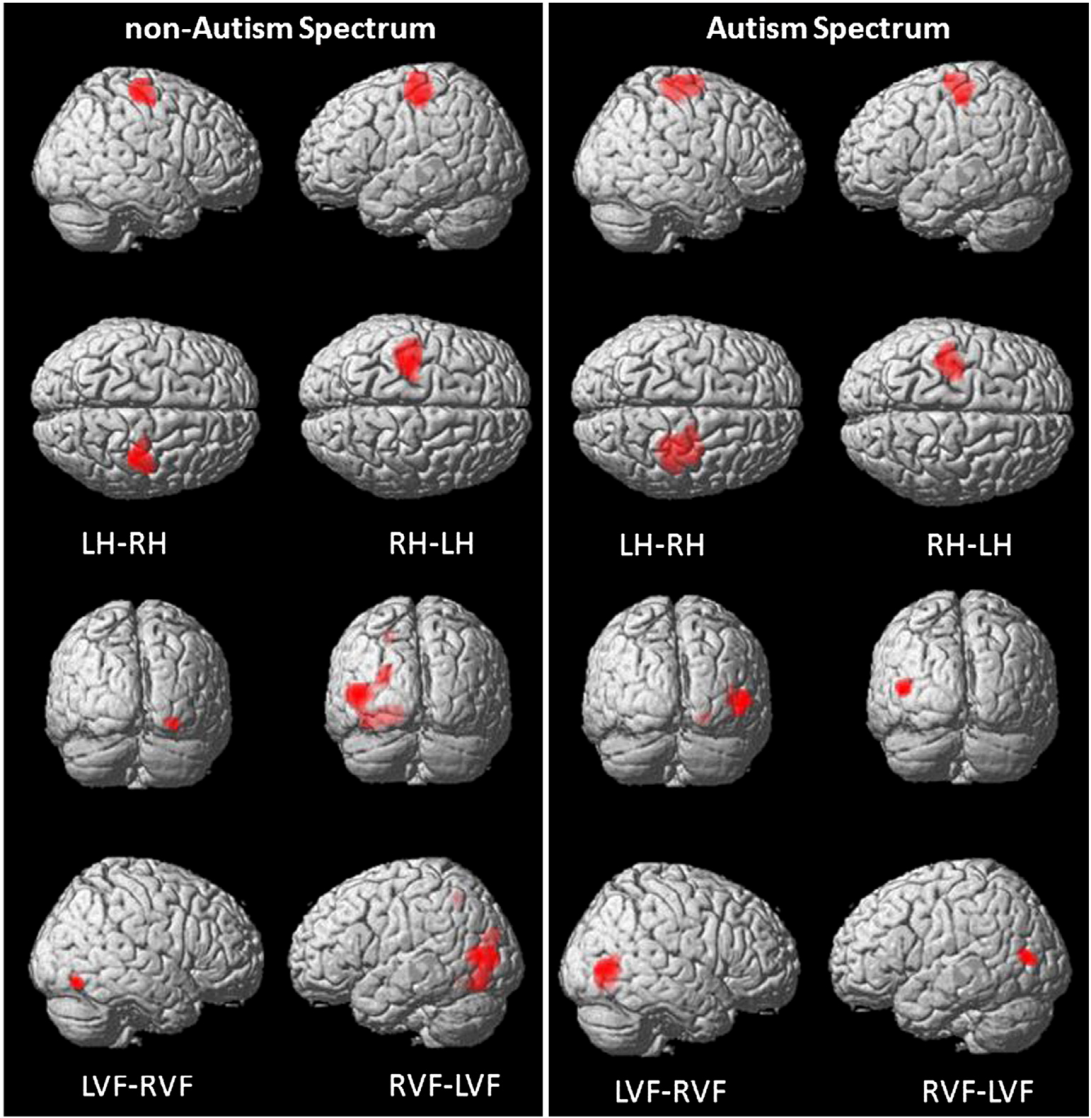
Within-group task-related activity patterns are displayed for the non-autism spectrum and the autism spectrum groups computed with the following contrasts: Main effect of hands (left hand minus right hand (LH − RH), right hand minus left hand (RH − LH)), and visual field (left visual field minus right visual field (LVF − RVF), right visual field minus left visual field (RVF − LVF)). Threshold: *t* = 4.96, *p* < .05, FWE, *k* = 50.

In order to investigate whether the AS individuals differed from the non-AS individuals in terms of the brain regions associated to the visuo-motor transfer of information, they performed an fMRI version of the Poffenberger paradigm. The regions activated during the Poffenberger task were similar between the two groups and included the left and right motor, premotor and visual cortices contralateral to the corresponding hand and visual field (Table 2, Fig. 6). This pattern of activation is consistent with other fMRI studies of the Poffenberger paradigm (e.g. Martuzzi et al., 2006; Tettamanti et al., 2002) and confirms that the visual stimuli were successfully presented to either the right or left hemisphere, and that the motor response was related to the contralateral motor cortical areas. These findings also confirm that the participants of both groups fixated adequately the center of the screen and that they carried out the requested motor response. There was no specific activation within or between groups for the Crossed conditions compared to the Uncrossed conditions. Patterns of activation were similar in both groups for the crossed conditions and the uncrossed conditions.

#### 3.4.2 Group differences in task-related brain activity

**Fig. 7:**
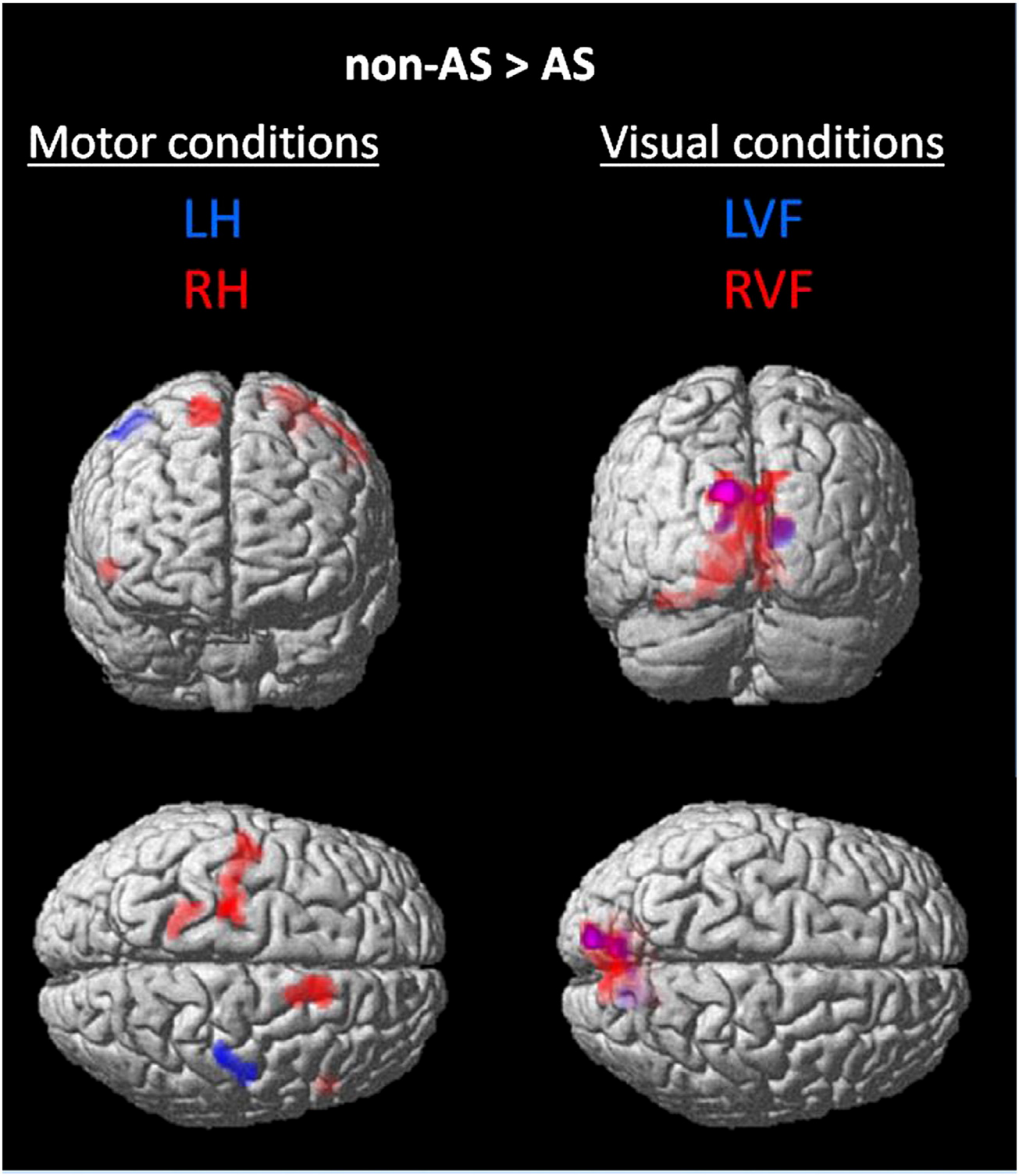
Areas of stronger activation in non-autistic (non-AS) than in autistic (AS) individuals are displayed. Group difference in task-related activity for the motor (left hand (LH) in blue, right hand (RH) in red (LVF and RVF pooled)) and visual (left visual field (LVF) in blue, right visual field (RVF) in red (LH and RH pooled)) contrasts. Threshold: *p* < .05, FWE, cluster correction *k* = 50.

The second level group analyses revealed a between-group difference: the visual and motor-related areas were more active in the non-AS than in the AS group (Table 2, Fig. 7). No areas were more active in AS than in non-AS individuals. For the RH-baseline contrast, the right supplementary motor area and left precentral gyrus were less active in AS than in non-AS individuals. For the LH-baseline contrast, the right precentral gyrus was less active in the AS than in the non-AS group. Overall, for both visual contrasts (LVF and RVF), the left and right visual areas were less active in AS than in non-AS individuals. This group difference was mainly driven by high activity in left occipital areas related to RVF stimulation, mostly for the RH_RVF condition, which is an uncrossed condition.

#### 3.4.3 Group differences in the lateralization of activation

Because more bilateral activity in homologous brain areas could be indicative of a more important IH communication, the magnitude of lateralization of bilateral activations was investigated. We conducted repeated measure ANOVAs separately for the voxel count and the voxel value measures of laterality index (LI), with Group as a between factor and Modality (motor: BA4, BA6, visual: BA17, BA18-19) as a within factor. There was a Modality × LI × Group interaction for the voxel count measure (*F*(3,30) = 3.05, *p* = .043). Task-related brain activity was more bilateral in the motor areas (mainly for the LH condition) in non-AS participants, whereas it was more bilateral in the visual areas in the AS participants. There was a group effect in the visual areas (*F*(4,30) = 2.92, *p* = .037), mainly in the primary visual areas, where bilateral activity was higher in AS than in non-AS individuals.

#### 3.4.4 Functional connectivity

**Fig. 8:**
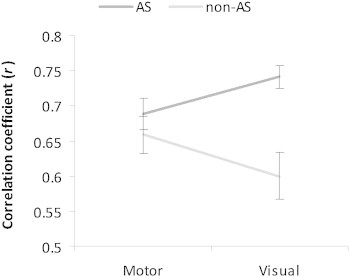
Correlation coefficients displayed for each group for the motor and visual ROIs. Error bars represent standard errors.

A repeated measure ANOVA on the correlation coefficient for each ROI pairs and participant revealed a Modality × Group interaction (*F*(1,38) = 7.577, *p* = .009). In the AS group, the results revealed a stronger connectivity between bilateral visual areas than between left and right motor ROIs, while the opposite was true for the non-AS group (Fig. 8). Post-hoc independent sample-*t*-tests did not reveal any group difference for the motor ROIs (*t*(39) = 0.901, *p* = .373), while the connectivity measures were significantly higher in the AS than in the non-AS group in the visual modality (Visual ROI 1: *t*(29.1) = 2.826, *p* = .008, visual ROI 2: *t*(32.9) = 3.763, *p* = .001, average *r* visual ROIs 1 + 2: *t*(28.5) = 3.748, *p* = .001).

### 3.5 Structure–performance relationship

We used exploratory regression analyses to investigate the existence of a relationship between the CC measures (size and DWI properties) and performance on the behavioral tasks and to examine whether this relationship (if any) differed between groups.

#### 3.5.1 Poffenberger

##### 3.5.1.1 Relative CC size

There was a Group × Relative CC size interaction for area 22–25 connecting the visual areas (*p* = .018). The size of the CC in these regions was associated with the IHTT in the AS group (*p* = .014), but not in the non-AS (*p* = .541) group. IHTT and CC size in the other four regions were not significantly correlated in either group. When all RelCC data (before the removal of outliers) were included in the model, there was a trend for the IHTT being correlated with RelCC 4_9 (*r* = .387, *p* = .083) and RelCC 22_25 (*r* = .385, *p* = .085) in AS individuals. In non-AS individuals, the IHTT was correlated with RelCC 4_9 (*r* = .602, *p* = .004) and with RelCC 17_21 (*r* = .385, *p* = .085).

##### 3.5.1.2 DWI measures

There was a Group × DWI measure interaction in the CC areas connecting the superior and middle occipital areas (FA_SO, FA_MO, RD_SO). The diffusion metrics (FA and RD) of the CC were associated with IHTT in AS participants (a high FA was associated with a low RD and a fast IHTT) but not in non-AS participants. No other regional CC property was associated with IHTT. Structure–performance relationships for the Poffenberger task are presented in Table 3.

#### 3.5.2 Purdue pegboard

There were significant Group × CC measure interactions; performance in the Purdue pegboard task was associated with the relative CC area of the posterior CC (Rel CC 22_25) in AS participants, but was associated with the more anterior (motor) CC properties (Rel CC 4_9, AD_SMA) in non-AS participants. The size of the CC in frontal regions was positively correlated with performance in non-AS individuals, whereas in AS individuals, the size of the CC in posterior areas was negatively correlated with performance. No significant associations were observed for the other regions. Structure–performance relationships for the Purdue pegboard task are presented in Table 4. When all RelCC data were included in the model (before the removal of outliers), performance in the Purdue BH task was negatively correlated with CC size in both parietal (*p* = .035) and occipital (*p* = .008) regions in AS individuals. Performance in the Purdue AS task tended to be correlated with RelCC 17_21 in non-AS individuals (*r* = .399, *p* = .066).

### 3.6 Summary of findings

We found that behavioral IHTT and performance in bimanual tasks were similar between AS and non-AS people, despite the fact that the CC in subregions connecting frontal and parietal cortical areas was smaller in the AS group. The size of the CC in subregions connecting posterior cortical regions as well as the diffusion measures were similar between groups. The primary motor areas contralateral to the hand used and the right supplementary motor area during tasks involving the right hand were less active in the AS group than in the non-AS group. The activity of visual areas was lower in AS participants than in non-AS participants during the presentation of stimuli in either the right or left visual field. Bilateral activity was highest in the motor areas in non-AS individuals whereas in AS individuals, it was highest in the visual areas. During the fMRI Poffenberger task performance, there was higher functional connectivity between bilateral posterior brain areas in the AS group than in the non-AS group. By contrast, activity in motor areas bilaterally was better correlated than activity in bilateral visual areas in the non-AS group. Behavioral IH performance was mainly correlated with the properties of CC subregions connecting bilateral motor areas in non-AS individuals, whereas it was associated with the properties of posterior CC regions in the AS group. We will now discuss the implication of these findings for the understanding of interhemispheric integration in AS people.

## 4 Discussion

### 4.1 Behavioral performance

Contrary to our prediction, neither the CUD computed from the Poffenberger task, nor bimanual performance in the Purdue task differed between autism spectrum (AS) and non-AS individuals. This suggests that the basic transfer of visuo-motor information is intact in adult AS individuals of typical intelligence, at least in AS individuals with speech delay and/or atypicalities (DSM-5) or autistic people (DSM-IV). This is consistent with the conclusion of Clawson et al. (2015), who used behavioral tasks and electrophysiological recordings in motor areas to investigate interhemispheric transfer in AS children. According to Barbeau et al. (2015), bimanual and coordination motor skills function normally in autistic individuals and motor deficits associated with autism may instead involve the speed of the execution of simple movements and response anticipation.

### 4.2 Structural and DWI corpus callosum measures

The regions of the corpus callosum (CC) connecting the frontal and parieto-temporal areas were smaller in AS than in non-AS individuals, but the size of the CC in sections connecting posterior regions was normal. Consistent with our findings, a meta-analysis of MRI studies of the CC in AS (Frazier and Hardan, 2009) concluded that the CC subregions connecting the premotor and supplementary motor areas are the most affected in AS, whereas those connecting visual areas display the smallest group differences. Similarly, Lazar and colleagues (2014) found no differences in any of the DTI metrics (FA, MD, RD or AD) of the CC between adult AS individuals of typical intelligence and a matched group of non-AS controls. The wide age range of the participants in our study (14–38) and that of Lazar et al. (18–25), more specifically the inclusion of adults, may account for the lack of difference between AS and non-AS individuals. Indeed, most studies examining the CC in AS have included younger participants (Travers et al., 2012). According to Kleinhans et al. (2012), differences in diffusion metrics of white matter between AS and non-AS individuals, are still present in adolescence and normalize in adulthood. In consequence the present results regarding diffusion metrics should not be generalized to younger AS populations in which group differences are more likely to be observed. Normalization into adulthood also occurs for brain volume (Amaral et al., 2008), and the symptoms of autism (Fecteau et al., 2003). The inclusion of AS participants of typical intelligence could thus also minimize the discrepancies between groups.

### 4.3 Task-related activation

There were no differences in cortical activation related to crossed or uncrossed conditions of the Poffenberger task within or between groups. A first generation of Poffenberger/fMRI studies in non-AS populations reported a correlation between CUD and the difference of signal intensity between the crossed and the uncrossed circuits (Iacoboni and Zaidel, 2004), with greater cortical activations in the crossed condition compared to the uncrossed condition (Tettamanti et al., 2002; Weber et al., 2005). However, four recent fMRI investigations of the Poffenberger paradigm reported that the crossed condition is not associated with specific patterns of activation (Gawryluk et al., 2009; Gawryluk et al., 2011; Martuzzi et al., 2006; Omura et al., 2004), other than the activation of white matter in the CC. However, the validity of the fMRI signal in white matter is questionable because the low energy consumption of axons and low blood volume and flow in white matter give rise to a weak signal (Gawryluk et al., 2009; Gawryluk et al., 2011). Differences in signal intensity between homologous cortical regions are informative as they may reflect deficits in IH information integration (Fornari et al., 2007). It is possible that all trials (crossed and uncrossed) recruit a common network with bilateral activations occurring even in the uncrossed conditions (Gawryluk et al., 2009; Martuzzi et al., 2006). This may explain the lack of significant difference in cortical BOLD signal between the crossed and uncrossed trials. Martuzzi et al. (2006) also suggested that the difference in lateralization of signal strength (e.g. a stronger signal in the right than in the left visual areas) could mask subtle differences specific to the crossed conditions, which include trials of both the left and the right VF stimulation.

We observed activation related to the main effects of the task (motor response, visual stimulation) in the left and right motor, premotor and visual cortices contralateral to the corresponding hand and visual field in both groups. There were few group differences. The activity during the right hand movement condition in the right BA6, the area opposite to the brain area controlling the right hand, was lower in AS than in non-AS individuals. The activity in the left and right BA6 areas was more bilateral in non-AS individuals whereas it was more bilateral in visual areas in AS individuals, which may be indicative of better integration between bilateral homologous motor regions in non-AS than in AS individuals (Fornari et al., 2007).

We also observed less task-related activity in visual areas in AS individuals. We expected the opposite result because most cognitive tasks involving visual input lead to high task-related activity in visual associative regions in autism (Samson et al., 2012). The comparatively low visual activity seen in AS individuals may be due to highly efficient perceptual processing, as observed in reasoning tasks in autistic individuals (Soulieres et al., 2009), in visuo-spatial tasks in non-AS individuals (Ruff et al., 2003), and in highly skilled non-AS individuals (Bernardi et al., 2013). Moreover, autistic individuals outperform non-AS individuals in visual tasks involving pattern manipulation but not in low-level detection tasks (Meilleur et al., 2014; Rivest et al., 2013), which may require shallower processing for detection.

### 4.4 Modification of the typical visual/motor balance contribution in visual/motor tasks in autism

Several studies have used variations of the Poffenberger paradigm, e.g. manipulation of visual (luminance, eccentricity) and motor (position of hand/arm) parameters to identify components affecting CUD and examine whether visuo-motor information crosses the CC at the motor (anterior) or visual (posterior) level. These studies, as well as partial lesions studies, have indicated that normal transfer takes place at the pre-motor level (Berlucchi et al., 1971; Clarke and Zaidel, 1989; Corballis et al., 2002; Tettamanti et al., 2002; Zaidel and Iacoboni, 2003). Lesions of the anterior CC sparing the splenium (posterior) cause abnormally long CUDs (Iacoboni and Zaidel, 1995; Marzi et al., 2003). However, long CUDs are also associated with posterior lesions (Marzi et al., 2003), and individuals with lesions of the CC (either anterior or posterior) may have normal CUDs (Berlucchi et al., 1995). These observations support the horse race model (Bisiacchi et al., 1994), in which visuo-motor information is transferred at both the pre-motor and visual levels. The CUD would reflect the “winning”, quickest, trans-callosal transfer which triggers the motor response. The fiber composition of the CC in typical individuals may support faster transfer through the motor fibers than through the visual fibers. The subregion connecting the sensori-motor cortical regions is composed of axons that are more myelinated and have a larger diameter (Aboitiz et al., 2003) than the axons connecting the visual cortices, which are longer (Lewis et al., 2009). Thus, conduction should be faster along motor fibers than along visual fibers. Moreover, in non-autistic individuals, microstructural properties (diffusivity and FA; Schulte et al., 2005) and size (Schulte et al., 2004) of the anterior CC (genu) correlate with the CUD.

The results of the present study suggest that the balance between the visual and motor contribution to the IH transfer of visuo-motor information is altered in autism. The functional connectivity analyses revealed a greater correlation of task-related activity between bilateral visual areas in the autistic than in the non-autistic group. Also, the balance of connectivity between bilateral motor and visual areas was reversed between the two groups; the connectivity being stronger in visual than in motor regions in autistics, while the opposite pattern was true for the non-autistic group. Moreover, IH transfer time was correlated with the relative CC size and diffusion properties of posterior regions in the autistic group but not in the non-autistic group. Similarly, in autistic individuals, there was a relationship between the properties of the posterior CC and the performance in a visuo-motor bimanual behavioral task, the Purdue pegboard, measured outside of the scanner, whereas performance in this task was related to the properties of the CC in motor-related areas in non-autistic individuals. Our results regarding bimanual performance in non-autistic individuals and its relationship with the properties of the anterior CC are also consistent with previous reports of (1) the relationship between the coordination of bimanual skills and CC integrity in the regions connecting the supplementary motor areas (Johansen-Berg et al., 2007); and (2) the relationship between CC size and fMRI activation of the cortical motor areas during a bimanual coordination task (Stancak et al., 2003).

Overall, this suggests that the transfer of visuo-motor information in non-autistic individuals involves the anterior, motor-dedicated CC, whereas in autism, its visual sections play a predominant role. In support of this interpretation, Lazar et al. (2014) reported that axonal and intra-axonal diffusivity across the CC and extra-axonal axial diffusivity localized in the anterior CC were lower in autistic than in non-autistic individuals. These metrics, obtained using diffusional kurtosis imaging, are reflective of axonal density and intra- and extra-axonal integrity, respectively. Moreover, performance in the Digit Symbol Coding test, a task that involves motor and visual functions as well as interhemispheric integration (Zaidel and Iacoboni, 2003), was correlated with the microstructural properties of the visual and temporal tracts in autistic individuals whereas it was related to the properties of the motor tracts in non-autistic individuals.

### 4.5 Plastic reorganization in autism

Autistic individuals performed well in tasks relying on IH transfer, despite the presence of a below average CC area and atypical task-related activation. This indicates an alternate structural and functional organization, and thus the relationship between behaviors and structure or activation in autistic individuals cannot be deduced from a direct comparison to the one observed in non-autistic people. Clawson et al. (2015) proposed that the performance of autistic individuals is normal in tasks involving IH transfer, despite anatomical alterations in the CC, due to the use of alternative neuronal pathways. Consistent with this view, we found that visual interhemispheric transfer plays an important role in visuo-motor tasks in autistic individuals. This observation also provides another example of the now well established enhanced role of perception in autistic cognitive architecture (see Mottron et al., 2014; Mottron et al., 2013 for recent reviews).

The modification of typical structure–function relationships in individuals showing a normal or superior level of performance in several visuo-spatial, intelligence and language tasks is consistent with the reallocation and extension of the role played by associative perceptual regions in such tasks. Cortical allocation in visual and motor associative regions displays high variability in autistic individuals (Poulin-Lord et al., 2014). A recent activation likelihood estimation (ALE) meta-analysis revealed topographical extension and reallocation of activity in visual associative regions during the completion of complex tasks involving visually presented material (Samson et al., 2012). For instance, a greater role of visual brain areas was observed in various cognitive tasks of non-perceptual nature such as reasoning (Soulieres et al., 2009) and language processing (Shen et al., 2012) tasks. Structural alterations of the CC, as well as of typical-task related brain activity, do not affect simple behavioral interhemispheric visuo-motor performance, because performance may be dependent on alternative regions in autistic people, mainly perceptual regions. This may, depending on the function, result in typical, superior or inferior performance.

## Figures and Tables

**Table 1:** Characteristics of participants in the autism spectrum (AS) and non-AS group for the behavioral study (outside the scanner) and the MRI study.

	Behavioral study			MRI study		
	AS	Non-AS	*p*	AS	Non-AS	*p*
N	32 (3F)	31 (3F)		22 (3F)	24 (3F)	
Age (SD)	21.5 (5.9)	21.5 (5.2)	.991	20.3 (5.5)	22.7 (5.3)	.149
Range	14–34	15–37		14–33	15–38	
FSIQ (SD)	99.8 (12.7)	106.7 (11.6)	.030	98.6 (10.7)	108.1 (13.0)	.010
Range	78–126	87–127		78–126	87–127	
PIQ (SD)	105.3 (11.3)	103.5 (11.87)	.555	104.7 (12.3)	106.1 (13.2)	.714
Range	77–127	82–122		77–127	82–122	
VIQ (SD)	96.3 (16.3)	108.5 (11.4)	.001	96.1 (15.0)	108.8 (12.2)	.003
Range	67–128	91–127		67–124	91–127	
RPM %tile (SD)	73.5 (22.2)	69.6 (23.7)	.508	70.4 (20.9)	72.1 (25.6)	.803
Range	10–100	25–98		10–100	25–100	
Edinburgh	81 (21.9)	77 (20.1)	.532	83 (20.5)	86 (11)	.640
Range	27–100	17–100		29–100	62–100	

**Table 2:** Activity associated with the visuo-motor Poffenberger task. Main effect of hands and visual fields are displayed for each group (autism spectrum (AS) and non-AS) as well as group differences. Each cluster's maxima is displayed in bold followed by the other significant peaks.

	BA	Left						Right					
		*x*	*y*	*z*	*t*	*d*	*k*	*x*	*y*	*z*	*t*	*d*	*k*
**NON-AS**													
**Motor contrasts**													
LH–RH													
*Post and precentral gyrus*	6, 4, 3, 1							**40**	**−24**	**64**	**8.27**	**2.00**	**330**
RH–LH													
*Post and precentral gyrus*	6, 4, 1	**−36**	**−22**	**66**	**10.32**	**2.83**	**699**						
**Visual contrasts**													
LVF–RVF													
*Fusiform, lingual gyrus*	18							**26**	**−70**	**−12**	**6.07**	**0.53**	**82**
RVF–LVF													
*Middle occipital gyrus*	19	**−46**	**−74**	**8**	**9.05**	**5.30**	**1647**						
*Lingual gyrus*	18	−20	−76	−8	7.64	0.62							
*Cuneus*	19	−26	−80	20	7.44	0.81							
*Superior parietal lobule*	7	**−20**	**−58**	**50**	**5.81**	**0.37**	**50**						

**AS**													
**Motor contrasts**													
LH–RH													
*Post and precentral gyrus*	6, 1, 4							**34**	**−24**	**70**	**8.29**	**2.39**	**643**
RH–LH													
*Post and precentral gyrus*	6, 4, 1, 3	**−38**	**−28**	**64**	**7.92**	**1.39**	**459**						
**Visual contrasts**													
LVF–RVF													
*Middle occipital gyrus*	19							**50**	**−66**	**2**	**7.01**	**0.63**	**460**
*Middle temporal gyrus*	39							42	−66	12	5.97	0.41	
*Lingual gyrus*	18, 19							**18**	**−76**	**12**	**5.50**	**0.08**	**73**
RVF–LVF													
*Middle occipital gyrus*	19	**−42**	**−74**	**10**	**6.66**	**0.57**	**144**						
**NON-AS > AS**													
RH													
*Precentral gyrus*	4,6	**−38**	**−22**	**64**	**7.62**	**1.55**	**328**						
*Supplementary motor area*	6							**10**	**18**	**64**	**7.23**	**1.22**	**152**
*Inferior frontal gyrus*	45							**52**	**20**	**−2**	**6.58**	**1.70**	**71**
*Postcentral gyrus*	5	**−18**	**−44**	**70**	**6.32**	**1.01**	**102**						
LH													
*Precentral gyrus*	4,6							**40**	**−26**	**62**	**7.19**	**1.03**	**123**
LVF													
*Superior occipital gyrus*	18,19	**−12**	**−92**	**30**	**6.48**	**1.09**	**181**						
*Calcarine gyrus*	17	−12	−78	14	6.20	0.52							
*Calcarine gyrus*	17							**16**	**−76**	**12**	**7.80**	**0.79**	**196**
*Cuneus*	18							14	−66	14	6.20	0.63	
RVF													
*Cuneus*	18,19	**−6**	**−80**	**26**	**7.68**	**0.96**	**2672**						
*Cuneus*	18							6	−74	24	6.96	0.92	
*Lingual gyrus*	18							8	−60	−2	7.00	0.63	

**Table 3:** Regression results showing the relationship between the Poffenberger interhemispheric transfer time (IHTT) measure and properties of the corpus callosum. Group X CC measure interactions and within-group effects are displayed for relative corpus callosum area for subregion 22 to 25 (Rel CC 22_25) as well as for fractional anisotropy (FA) in the corpus callosum connecting the superior occipital areas (FA_SO), the middle occipitals areas (FA_MO) and the radial diffusivity for the superior occipital regions (RD_SO). There was no group effect or interaction for any other corpus callosum measure or subregion.

	CC measure	Group × CC measure interaction	Within-group effects	
			Non-AS	AS
*Visual*	RD_SO	*R* = .373, *t*(1,35) = 1.973, *p* = .056	*R* = −.035, *p* = .885	***R* = −.480, *p* = .038**
	FA_SO	*R* = .426, *t*(1,37) = 1.971, *p* = .056	*R* = −.082, *p* = .725	***R* = −.543, *p* = .013**
	FA_MO	*R* = .454, *t*(1,37) = 2.683, *p* = .011	*R* = .077, *p* = .739	***R* = −.580, *p* = .007**
	Rel CC 22_25	*R* = .451, *t*(1,36) = −2.475, *p* = .018	*R* = −.145, *p* = .541	***R* = .540, *p* = .014**

**Table 4:** Regression results showing the relationship between the Purdue pegboard “Both hands” (BH) and “Assembly” bimanual conditions and properties of the corpus callosum. Group X CC measure Interactions and within-group effects are displayed for relative corpus callosum area for subregion 4 to 9 (Rel CC 4_9), 17 to 21 (Rel CC 17_21) and 22 to 25 (Rel CC 22_25) as well as for axial diffusivity in the corpus callosum connecting the supplementary motor areas (AD_SMA). There was no group effect or interaction for any other corpus callosum measure or sub-region.

	CC measure	Purdue	Group × CC measure interaction	Within-group effects	
				Non-AS	AS
*Motor*	Rel CC 4_9	BH	*R* = .388, *t*(1,35) = 2.09, *p* = .044	***R* = .405, *p* = .076**	*R* = .292, *p* = .224
		Assembly	*R* = .322, *t*(1,35) = 1.59, *p* = .122	***R* = .429, *p* = .059**	*R* = .140, *p* = .569
	AD_SMA	BH	*R* = .395, *t*(1,36) = 1.95, *p* = .059	***R* = .459, *p* = .042**	*R* = .012, *p* = .960
		Assembly	*R* = .451, *t*(1,36) = −1.38, *p* = .177	***R* = .558, *p* = .011**	*R* = .313, *p* = .179
*Parietal*	Rel CC 17_21	BH	*R* = .487, *t*(1,37) = 3.09, *p* = .004	***R* = .381, *p* = .088**	***R* = .553, *p* = .011**
		Assembly	*R* = .341, *t*(1,37) = 1.87, *p* = .069	***R* = .444, *p* = .044**	*R* = .197, *p* = .405
*Visual*	Rel CC 22_25	BH	*R* = .176, *t*(1,37) = 2.23, *p* = .032	*R* = .185, *p* = .423	***R* = .549, *p* = .012**
		Assembly	*R* = .333, *t*(1,37) = 1.95, *p* = .059	*R* = .170, *p* = .460	***R* = .433, *p* = .057**
