# Supplementary material for: A greater involvement of posterior brain areas in interhemispheric transfer in autism: fMRI, DWI and behavioral evidences
Source: Neuroimage Clin. 2015 Apr 30;8:267–80. doi: 10.1016/j.nicl.2015.04.019 (PMC4474173; doi:10.1016/j.nicl.2015.04.019)
Supplement: Supplementary file 1 — Supplementary material [file mmc1.doc]

**Supplementary figures**


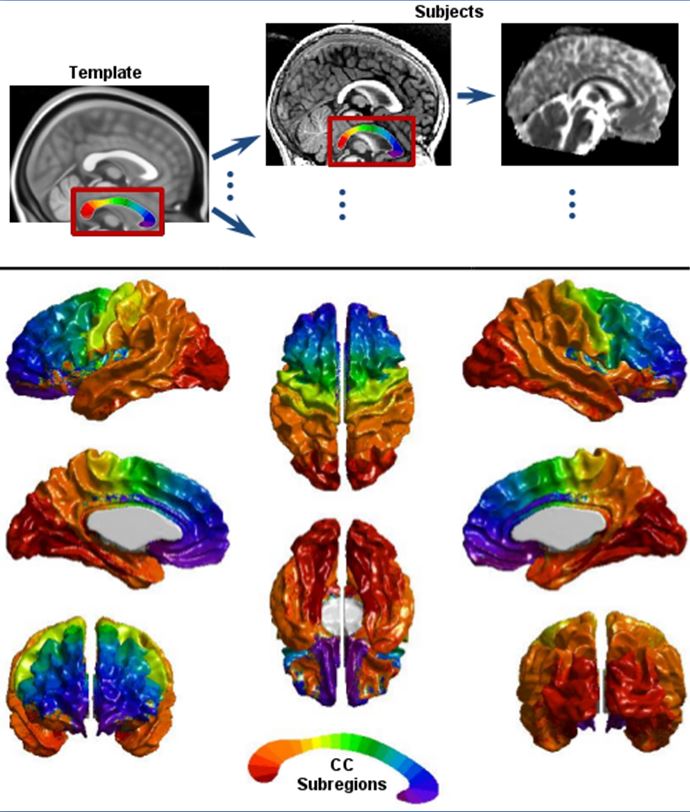


**Figure Supp 1**. Mapping of surface vertices to the 25 CC subregions with colors corresponding to the CC subregion at which tracts originating from each vertex most often terminated across all subjects. From Lewis et al. 2013 with permission.

**
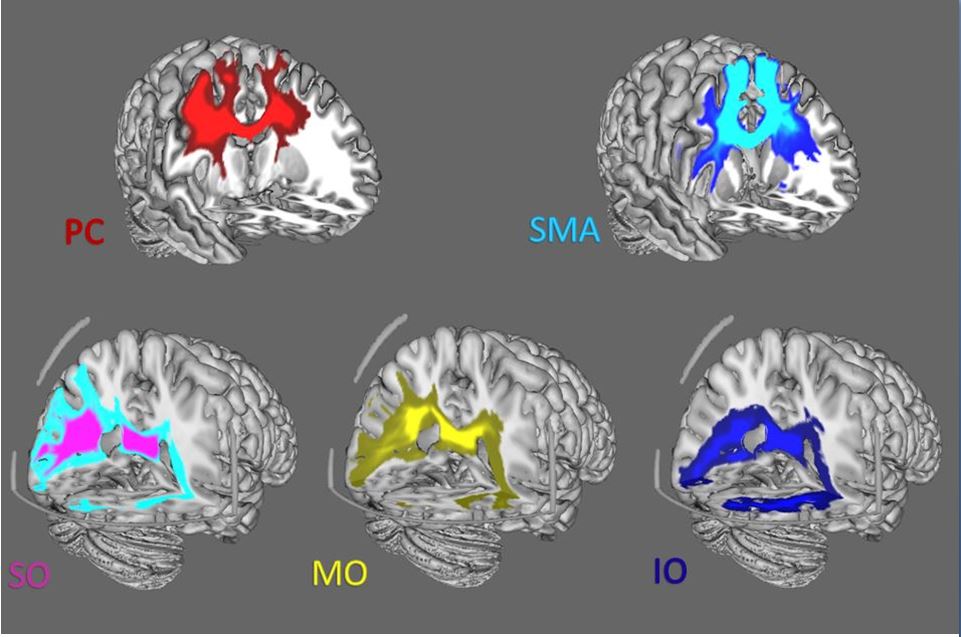
**

**Figure Supp 2.** Probabilistic tractography maps from 93 subjects in the ICBM dataset for the five ROIs: Precentral gyrus (PC), Supplementary motor area (SMA), Superior occipital gyrus (SO), Middle occipital gyrus (MO) and Inferior occipital gyrus (IO).

**
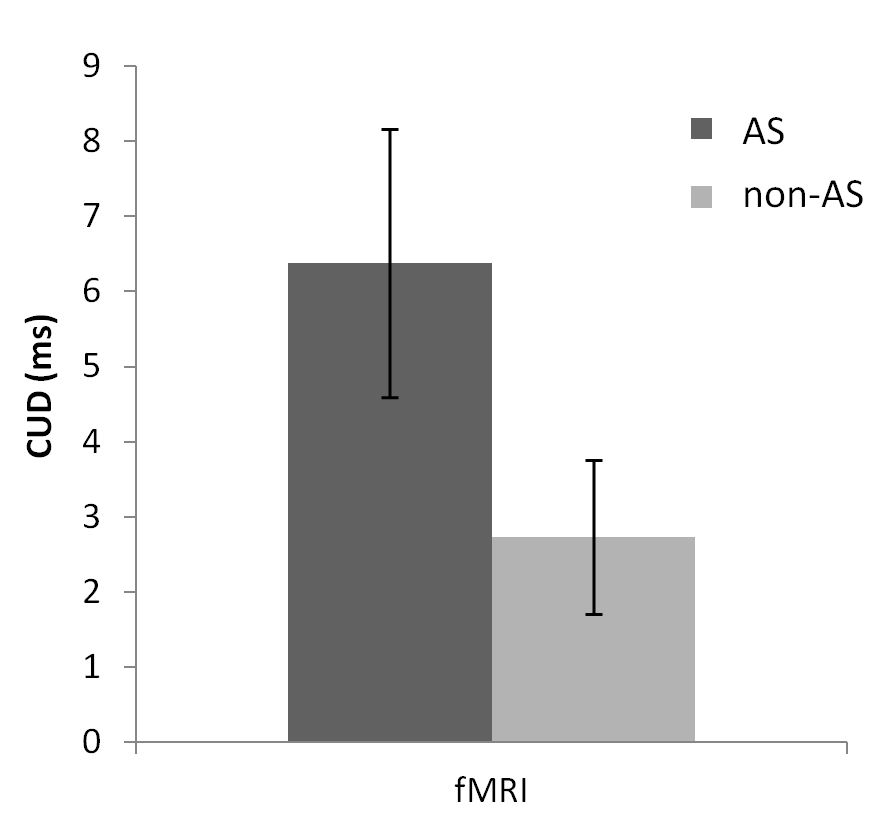
**

**Figure Supp 3.** Results of the Crossed-Uncrossed Difference (CUD) in milliseconds (ms) for the Autism Spectrum and non-AS groups measured with the Poffenberger task inside (fMRI) the scanner.

**
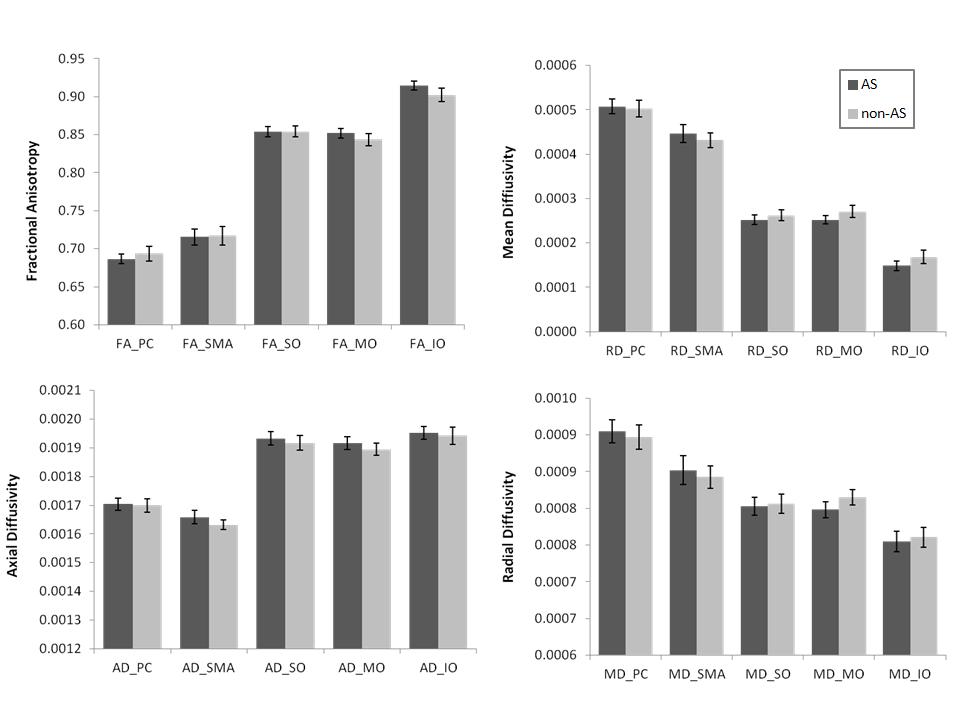
**

**Figure Supp 4**. Fractional anisotropy (FA), mean diffusivity (MD), axial diffusivity (AD) and radial diffusivity (RD) for the regions of interest of the corpus callosum connecting the bilateral pre-central (PC), supplementary motor (SMA), superior occipital (SO), middle occipital (MO) and inferior occipital (IO) cortical areas. Values are displayed for the Autism Spectrum (AS) and non-AS groups.
